# Real-Time Measurement of Herbicides in the Atmosphere: A Case Study of MCPA and 2,4-D during Field Application

**DOI:** 10.3390/toxics7030040

**Published:** 2019-08-06

**Authors:** Trey Murschell, Delphine K. Farmer

**Affiliations:** Department of Chemistry, Colorado State University, Fort Collins, CO 80523, USA

**Keywords:** atmospheric chemistry, pesticides, field, chemical ionization, mass spectrometry

## Abstract

Atmospheric sources of herbicides enable short- and long-range transport of these compounds to off-target areas but the concentrations and mechanisms are poorly understood due, in part, to the challenge of detecting these compounds in the atmosphere. We present chemical ionization time-of-flight mass spectrometry as a sensitive, real-time technique to detect chlorinated phenoxy acid herbicides in the atmosphere, using measurements during and after application over a field at Colorado State University as a case study. Gas-phase 2,4-dichlorophenoxyacetic acid (2,4-D) mixing ratios were greatest during application (up to 20 ppt_v_), consistent with rapid volatilization from spray droplets. In contrast, atmospheric concentrations of 2-methyl-4-chlorophenoxyacetic acid (MCPA) increased for several hours after the initial application, indicative of a slower source than 2,4-D. The maximum observed gas-phase MCPA was 60 ppt_v_, consistent with a post-application volatilization source to the atmosphere. Exposure to applied pesticides in the gas-phase can thus occur both during and at least several hours after application. Spray droplet volatilization and direct volatilization from surfaces may both contribute pesticides to the atmosphere, enabling pesticide transport to off-target and remote regions.

## 1. Introduction

Herbicides are widely used throughout the United States, with up to 2.5 billion pounds of total herbicide applied annually [1]. The off-target effects from aquatic transport and soil run-off of pesticides are the typical focus of environmental studies, but the sources and fate of pesticides in the atmosphere also impact the ecosystem and human health. Atmospheric concentrations cause unintentional exposure to agricultural workers and pollinator populations [2,3,4,5,6]. Atmospheric transport enables deposition to off-target areas (e.g., neighboring agricultural sites via short-range transport and more distant areas via long-range transport) [7,8,9,10,11,12,13,14,15], and is controlled by atmospheric chemistry, including gas/particle partitioning, oxidation chemistry, and removal by wet or dry deposition. However, investigation of atmospheric pesticide sources and fate requires fast and sensitive instruments.

Atmospheric pesticide measurements are typically restricted to off-line sample collection and subsequent analysis, often following time- and solvent-consuming extractions, with sample collection times ranging from hours to days [16,17,18]. For example, high volume samplers pass hundreds of cubic meters of air through filters in the space of 24 h, enabling sample concentration for subsequent analysis. These actively-collected filters and sorbents, such as polyurethane foam or XAD-2 [19], are then manually changed to obtain time-resolved sampling. Alternately, passive samplers comprising absorbent material can be placed in outdoor environments, but typically require much longer (days to weeks at best, often on the order of two to three months for seasonal sampling) sample collection time and are not typically used for pesticides that partition between gas and particle phases [20]. Off-line analysis of filters or adsorbent cartridges typically involves gas or liquid chromatography coupled to mass spectrometry and can be adequately sensitive for ambient measurements, even in remote locations. However, recent developments in field-portable mass spectrometry enable much faster measurements and offer the potential to directly measure gas-phase pesticides in the atmosphere [21,22].

Phenoxy acids are a class of herbicides that include 2,4-dichlorophenoxyacetic acid (2,4-D). 2,4-D is toxic to humans and is linked to skin problems, cancer, and reproductive issues [23,24,25]. 2,4-D is often paired with other herbicides in commercial mixtures, including 2-methyl-4-chlorophenoxyacetic acid (MCPA) and other chlorinated aromatic alkyl acids. Few atmospheric measurements of these 2,4-D-type compounds exist. A four-year study of phenoxy acid herbicides in Canada showed gas-phase atmospheric 2,4-D and MCPA concentrations on the order of micrograms per cubic meter [26], although higher concentrations have been observed in the agricultural prairie regions (0.11–2.73 ng/m^3^ [0.014–0.355 ppt_v_, STP] for 2,4-D and 0.17–1.88 ng/m^3^ [0.017–0.190 ppt_v_] for MCPA) [27]. 2,4-D and MCPA are currently used in both agriculture and commercial applications. Like many university campuses, Colorado State University sprays herbicides annually to maintain campus grounds and protect from broadleaf weeds. This annual spraying provides a test case that represents, to the best of our knowledge, the first real-time measurements of herbicides in the atmosphere during and after application.

Here, we describe the application of time-of-flight chemical ionization mass spectrometry with acetate ionization to the direct measurement of atmospheric pesticides in the field. These measurements highlight the different mechanisms for pesticide release into the atmosphere, and the potential for rapid measurements to track the sources and fate of these anthropogenic pollutants. This work is intended to be a case study, rather than a generalizable or quantifiable description of pesticide sources or fate to the atmosphere. However, these measurements highlight the potential for real-time measurements during application to study drift and other processes.

## 2. Materials and Methods

### 2.1. Field Measurements

Commercially-available herbicide mixtures were applied between 6:42 and 7:08 am (local time, MST) on 3 September 2017 to a 2000 m^2^ plot of grass adjacent to the Chemistry building on the main campus of Colorado State University (Fort Collins, CO, USA). On the morning of sampling, weather records show that the wind was 5 km/h from the north; temperatures were at a low of 14 °C at 6 am and reached 34 °C by noon. Average relative humidity between 6 am to noon (local time) was 40%, and the average pressure was 1017 mbar. The application target was a campus lawn comprising well-watered turf, with concrete buildings to the south, east, and west. To the north, the campus includes roads, paths, and other buildings. As the application discussed herein was the first, and to our knowledge only, herbicide application on the campus on this day, this application can be considered the main local source at this site.

The applied herbicide mixture included Battleship 3 (Helena Agri-Enterprises, LLC, Collierville, TN, USA; includes MCPA, triclopyr, and fluroxypyr) and HardBall (Helena Agri-Enterprises, LLC, Collierville, TN, USA; includes 2,4-D). Battleship 3 is typically marketed on its lack of 2,4-D for use in sensitive regions, but in this case, the Colorado State University (CSU) Facilities purposely added 2,4-D to maximize efficacy. The net mixture was representative of best practices for commercial application and standard for campus application. The active ingredients in the mixture specifically included 2,4-D, the dimethylamine salt of MCPA, the triethylamine salt of triclopyr (3,5,6-trichloro-2-pyridinyloxyacetic acid), and the methylheptyl ester of fluroxypyr (4-amino-3,5-dichloro-6-fluoro-2-pyridyloxy-acetic acid, 1-methylheptyl ester). The herbicide spray solution was prepared in an 1136 L drum according to the manufacturer’s specifications: 9.4 L of commercial 2,4-D (19.6% by weight; 208 g/L 2,4-dichlorophenoxyacetic acid on an isomer specific basis) and 11 L of a commercial mixture of MCPA, triclopyr, and fluroxypyr (37.84%, 4.07%, 4.45%, by weight of the original commercial mixture, respectively; 340 g/L 2-methyl-4-chlorophenoxyacetic acid/MCPA, 35 g/L 3,5,6-trichloro-2-pyridinyloxyacetic acid/triclopyr, 32 g/L ((4-amino-3-5-dichloro-6-fluoro-2-pyridinyl)oxy)acetic acid/fluroxypyr on an isomer specific basis) were added to the drum; water was added to fill the remaining volume.

The herbicide mixture was applied by the standard approach used on the CSU campus, but only for a short period (total application took < 30 min). A 4 m boom sprayer pulled by a small tractor applied the herbicide mixture. At the site, the applicator sprayed a total of ~64 L of the herbicide solution 0.3 m from the ground. The application covered a circular pattern, in which each successive loop was smaller, ~4 to 5 m closer towards the center of the plot.

**Figure 1:**
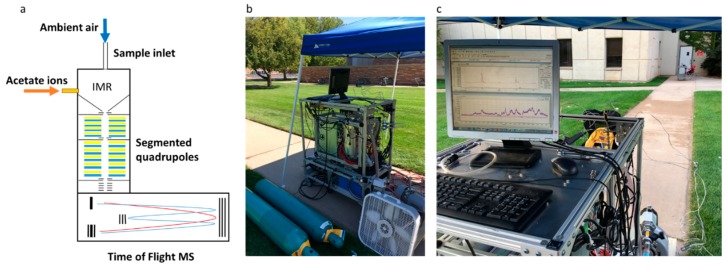
(**a**) A schematic of the time-of-flight chemical ionization mass spectrometer with acetate reagent ions (acetate-CIMS) demonstrating how ambient air is pulled in from a vertically-oriented inlet into an ion-molecule reaction chamber (IMR), where ambient air molecules are ionized through reaction with acetate ions. Air and ions are pulled through a series of segmented quadrupoles as the pressure is dropped, before orthogonal extraction into a time-of-flight detector; (**b**,**c**) The acetate-CIMS was located under a shaded tent. Pesticides were applied to the surrounding lawn.

We sampled the ambient atmosphere in real-time (data collected at 1 Hz, averaged in post-processing to 10 s) before, during, and after the application. Gas-phase mixing ratios were measured by a time-of-flight chemical ionization mass spectrometer with acetate reagent ions (hereafter referred to as acetate-CIMS). The acetate-CIMS was located on a paved footpath in the middle of the application area (Figure 1). At its innermost loop, the booms passed ~1 m from the instrument inlet. No further herbicide application was conducted in the vicinity of the measurement area after 7:08 am to avoid confusing drift with volatilization.

We emphasize that the patchwork nature of campus lawns requires a different method of application from large agricultural regions, limiting the generalizability of these observations. Further field measurements will be essential to accurately quantify herbicide sources to the atmosphere from pesticide application over different land-use types. That said, this work aims to demonstrate the potential for real-time gas-phase herbicide measurements in the atmosphere and provide evidence that herbicide application can be a source of atmospheric toxins.

### 2.2. Acetate-CIMS

The acetate-CIMS (Tofwerk AG, Thun, Switzerland and Aerodyne Research, Inc., Billerica, MA, USA) is described in detail elsewhere [28,29]. The acetate-CIMS is a real-time mass spectrometer designed for atmospheric chemistry measurements. Our instrument, in particular, is racked to ensure it is portable (300 kg; 0.6 m × 1.1 m × 1.3 m; on wheels; power requirements < 2 kW), enabling field measurements [28,30,31,32]. The acetate-CIMS consists of a short inlet (<5 cm of stainless steel) that samples ambient air into an ion-molecule reaction chamber. Ambient air is mixed with acetate reagent ions at decreased atmospheric pressure (typically 80–100 mbar), before progressing through a series of segmented quadrupoles as the pressure is reduced before the time-of-flight detector. 

During this study, the inlet was oriented vertically, sampling 1.3 m above ground level. This orientation and setup ensured that spray droplets released near ground-level at the base of the tractor at 0.3 m above ground level would not directly enter the inlet, thereby minimizing interferences. Observed herbicides must, therefore, enter the instrument from over one meter above the application point. Ambient air was pulled into the instrument at 1.9 standard L min^−1^ into an ion-molecule reaction chamber (IMR, 100 mbar, 40 °C), where molecules in the air were ionized by acetate reagent ions. Acetate ions were generated by passing acetic anhydride through a ^210^Po ionizer. The mixture of air and reagent ions was directed through two segmented quadrupole regions as the pressure in the mass spectrometer dropped to 10^−6^ mbar. Ions were pulsed orthogonally into a time-of-flight mass spectrometer. The acetate-CIMS was tuned to negative ion mode, with a range of *m*/*z* 4–500, mass accuracy < 20 ppm, mass resolution (*m*/Δ*m*) > 4000, and time resolution of 1 s. The high mass resolution of the TOF enabled ion elemental composition analysis. 

Acetate ionization occurs by either deprotonation [29], or by a clustering reaction in the IMR that can be coupled to a declustering reaction in the segmented quadrupoles. The net reaction is typically:

H_3_C_2_O_2_^−^ + HM → H_3_C_2_O_2_H + M^−^ (R1)

Because the gas-phase basicity of acetic acid is so high, acetate-CIMS is extremely sensitive to organic acids and is well-suited for the phenoxy acid herbicides [28,32,33]. We calibrated the acetate-CIMS in the laboratory with heated solution injections of 2,4-D and MCPA standards (US EPA Pesticide Repository, 99%) diluted in methanol (HPLC grade, Sigma Millipore, Burlington, MA, USA) [21]. 2,4-D is observed as a deprotonated molecular ion (C_8_H_6_Cl_2_O_3_^−^, *m*/*z* 218.96), with a minor contribution from a fragment ion (C_6_H_4_Cl_2_O^−^, *m*/*z* 160.95). The relative ion signal for 2,4-D is 2:1 for C_8_H_6_Cl_2_O_3_^−^: C_6_H_4_Cl_2_O^−^. MCPA and triclopyr are also observed as a deprotonated molecular ion (C_9_H_8_ClO_3_^−^ for MCPA; C_7_H_3_Cl_3_NO_3_^−^ for triclopyr) with negligible contributions of fragment ions. These peak assignments were verified by the presence of high-resolution ion peaks corresponding to isotopes in signals proportional to natural abundance. The sensitivity of the acetate-CIMS is 1.3 ± 0.4 and 1.5 ± 0.4 normalized counts ppt_v_^−1^ (parts per trillion by volume) for deprotonated molecular ions of 2,4-D and MCPA, corresponding to detection limits (3σ/sensitivity) of 3.1 ppt_v_ and 12 ppt_v_ in 1 s averaging, respectively. 

We overflowed the inlet with Ultra High Purity zero air (AirGas, total hydrocarbon content <0.01 ppm) each hour for 2 to 5 min to quantify the instrument background. Data collection began at 06:30 am, and continued until 11:30 am, when ambient temperatures reached 32 °C, and the temperature on the TOF detector itself reached 40 °C, as the instrument was not in an enclosed or air-conditioned structure. 

### 2.3. Data Analysis 

We analyzed data using Tofware (v2.5.6, Tofwerk AG, Thun, Switzerland) and Igor Pro (v6.37, Wavemetrics, Portland, OR, USA, 2015). We fit mass spectral peaks that corresponded to the applied herbicides (targeted analysis). We also fit all mass spectral peaks that met an enhancement criterion (i.e., the ratio of any signal during the closest pass of the herbicide applicator to the pre-spray period ≥3 through non-targeted analysis). The targeted analysis identified peaks corresponding to 2,4-D and MCPA, but not triclopyr or fluroxypyr. Multiple lines of evidence validated the assignment of mass spectral peaks to 2,4-D and MCPA. Each identified ion has multiple isotopes, and signals for those isotopes were proportional to expected natural abundances. NO_2_^−^ and NO_3_^−^ are strong tracers of diesel exhaust, [34] and displayed little correlation with the pesticide signals (*r*^2^ ≈ 0.1), confirming that the signals attributed to herbicides were not from the tractor exhaust. Finally, four zero air overflow experiments throughout the field experiment demonstrated that ambient signals were substantially higher than background signals from the instrument itself, confirming that the herbicide signal was truly ambient, and neither variable instrument background nor inlet artifact.

## 3. Results

**Figure 2:**
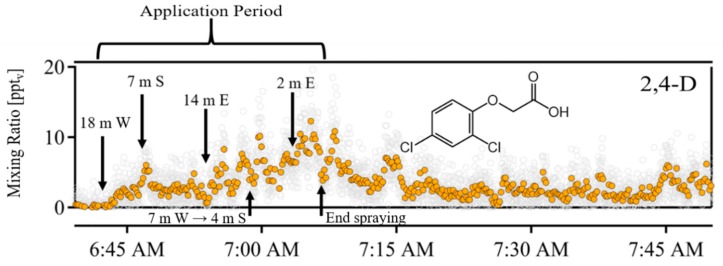
Time series of 2,4-dichlorophenoxyacetic acid (2,4-D) (upper panel, detected as a deprotonated molecular ion *m*/*z* 218.96) mixing ratios before, during, and immediately after the application period. Grey circles represent the 1 s data points, while colored circles are the 10 s averages. Application began at 6:42 am local time and ended at 7:08 am; each applicator pass is annotated on the time series of 2,4-D with the distance of the tractor (e.g., 18 m), and the direction of closest point of the applicator (East, West, South of the acetate-CIMS). Decreases in signal during the application period corresponded to periods when the spray boom was briefly turned off.

**Figure 3:**
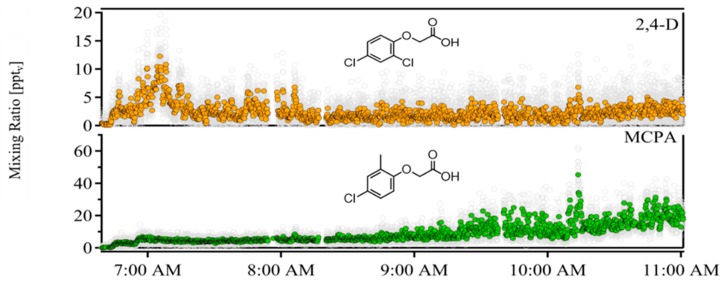
Time series of 2,4-D (upper panel) and MCPA (lower panel) during the entire measurement period. Ambient temperature rose from 15 °C to 35 °C at the conclusion of the measurement period.

Herbicide application caused clear increases in both 2,4-D and MCPA mixing ratios above background ( Figure 2 and Figure 3). No herbicide-related peaks were observed before application. Once spraying commenced, peaks associated with 2,4-D were immediately apparent, reaching a maximum of 13 ppt_v_ during the closest pass of the spray boom, with an average of 4 ± 3 ppt_v_ (1σ) during the application period. MCPA mixing ratios were also enhanced above background during the application period (average 4 ppt_v_), but reached their maxima several hours after application (70 ppt_v_ at 10:15 am). Neither fluroxypyr nor triclopyr was observed in the field; the acetate-CIMS instrument is capable of detecting these herbicides in lab settings, but these compounds were an order of magnitude lower in concentration than MCPA in the applied solution, suggesting that the concentrations were below the instrument’s detection limit.

## 4. Discussion

The Acetate-CIMS was adequately sensitive to observe at least two gas-phase phenoxy herbicides (2,4-D and MCPA) during and after application with fast (1 s) time resolution. To the best of our knowledge, such field measurements of pesticides in the atmosphere are unprecedented. Atmospheric measurements of these herbicides are typically limited to a much longer time resolution of hours or days and require sample collection followed by work-intensive off-line analysis. For example, high volume air samplers with filter collection for 2 to 12 days integration times before laboratory analysis by GC-MS with detection limits in the order of 1 to 5 pg/m^3^ for MCPA and 2,4-D for a 350 m^3^ sample collection, corresponding to a 24-h integration of the sampler. In contrast, the acetate-CIMS detection limits of 12 and 3.1 ppt_v_ reported herein for MCPA and 2,4-D, respectively, correspond to 0.099 and 0.027 μg/m^3^ in 1 second. Obviously, longer integration times lower the acetate-CIMS detection limit substantially. Thus, while acetate-CIMS will not be competitive for monitoring of remote locations, this work clearly demonstrates its utility for near-application measurements. To place these numbers in perspective, in their study of air concentrations of pesticides in Canada, Yao et al. [35] noted maximum air sample concentrations of 4.96 ng MCPA m^−3^ and 0.897 ng 2,4-D m^−3^ (1 m above ground level) during application periods in agricultural areas of Saskatchewan during spring and summer 2003, although averages were 0.5 and 0.2 ng m^−3^ for MCPA and 2,4-D, respectively. Thus, while the sensitivity of acetate-CIMS is likely insufficient for eddy covariance flux analysis, longer averaging times of 1 or 5 min should yield detection limits adequate for regional agricultural studies during periods of application. The rapid and moderately sensitive measurements are adequate for field applications, such as investigating airborne pesticide drift during and immediately after application by measurements immediately adjacent to application areas, as well as laboratory measurements to determine chemical kinetics and oxidation chemistry [36,37].

Application of phenoxy herbicides caused elevated mixing ratios of two confirmed compounds during and after application. The magnitude and timing of the atmospheric enhancement varied by compound, indicative of different atmospheric source mechanisms and emphasizing the difference in applied herbicide mixture from observed atmospheric concentrations. Application spray releases aqueous droplets that are large enough to rapidly deposit, and are thought to contribute to off-target effects over only short distances through spray drift. However, while spray droplets are in the atmosphere, organic compounds can volatilize directly from spray droplets, forming a spray drift volatilization source of gas-phase pesticides to the atmosphere during the application process. Herbicide volatilization from soil or water surfaces to the atmosphere is better studied than spray drift volatilization [17,38]. Short-term volatilization directly from ground and plant surfaces is considered well-described by vapor pressure [4]. Once pesticides have penetrated the soil, volatilization is slower (long-term volatilization), and is typically described by a combination of vapor pressure, Henry’s law constant, soil adsorption, and water solubility. Compounds with lower Henry’s Law constants are more mobile in soil, and can thus accumulate at soil surfaces and volatilize [39,40,41]. However, recent studies suggest that vapor pressure is the main predictor of volatilization, while other physiochemical properties only change the effectiveness of vapor pressure [42,43]. However, these models focus on long-term volatilization over tens of days, rather than short-term volatilization immediately after application. The time-resolved atmospheric herbicide measurements presented in this case study provide insight into the potential importance of short-term volatilization in the hours after application.

Two atmospheric sources of herbicides were possible during these experiments: (1) spray drift volatilization from herbicides in spray droplets partitioning to the gas-phase and (2) short-term volatilization from direct partitioning of the applied mixture on soil and plant surfaces to the atmosphere. Herbicides that have permeated into soil require longer (days to weeks) timescales for volatilization because these compounds react with soils and require time to transport back to the soil surface for subsequent volatilization.

Mixing ratios of 2,4-D were highest during the application process, indicating that spray volatilization was likely the dominant atmospheric source of both compounds. A spray volatilization source of MCPA was also apparent: gas-phase mixing ratios were elevated above background during the application process. However, spray volatilization was not the dominant atmospheric source of MCPA: mixing ratios maximized after application ceased. These results are consistent with the extent of spray volatilization being described by the Henry’s Law constant, which describes the ratio of gas-phase to aqueous concentration at equilibrium (Table 1). To the best of our knowledge, these are the first field measurements to suggest that spray volatilization could be a substantial source of gas-phase pesticides to the atmosphere.

Post-application volatilization was apparent for both 2,4-D and MCPA, but dominated by MCPA (Figure 3). Post-application MCPA mixing ratios were well-correlated to calculated vapor pressure (*r* = 0.75), consistent with the evaporation of this herbicide from applied solutions. Post-application 2,4-D is less correlated with temperature (*r* = 0.6).

Gas-phase pesticides have three potential environmental impacts: (1) direct inhalation and exposure to people and off-target ecosystems in the local area, (2) oxidation or other chemistry to form secondary air pollutants, and (3) atmospheric transport to off-target and remote areas. Observed atmospheric mixing ratios of the two herbicides were well below reported LD_50_ levels for acute exposure via inhalation (1.79 and 6.3 mg/L, ~100 and 700 ppm_v_ for 2,4-D and MCPA in rats, respectively), although sub-lethal health effects are possible, and correlations between phenoxy acid herbicide exposure and Non-Hodgkin’s lymphoma have been observed in agricultural workers [50,51]. Our observations imply that applicators should continue to wear personal protective equipment when working in an affected area for several hours after application. Of course, phenoxy herbicides are not the only pesticides subject to spray droplet volatilization and direct volatilization: Other compounds with comparable Henry’s Law constants and vapor pressures may also have substantial atmospheric sources during and after application. Several pesticides, including chlorpyrifos, acephate, and diazinon, have lethal and sub-lethal effects on bees; the chemical properties of those molecules (Henry’s law constants of 4.16 × 10^−6^, 5.2 × 10^−13^, 0.112 × 10^−6^ atm m^3^ mole^−1^ and vapor pressures of 2.2, 0.17, and 9.01 × 10^−5^ mmHg, respectively) suggest that atmospheric sources could contribute to pollinator exposure [52,53,54].

Anthropogenic organic compounds, including the phenoxy herbicides described herein, can be oxidized by OH radicals and other oxidants in the atmosphere [36,37]. The calculated OH reactivity from observed concentrations and known rate constants for the two herbicides is small, <0.01 s^−1^ with MCPA dominating the reactivity. This small contribution is unlikely to contribute to ground-level ozone production in the Front Range region of Colorado, where OH reactivity is typically 1 to 3 s^−1^ [55]. However, the spray solution used by CSU was formulated and diluted to minimize volatilization, while larger agriculture operations may spray more concentrated solutions less judiciously. Thus, while the atmospheric sources had a minor effect on ozone production in the local area, volatilization from larger-scale applications could impact local air quality. Oxidation products from phenoxy herbicides include an array of peroxide, carbonyl and carboxylic acid moieties, and may contribute to secondary organic aerosol [36].

## 5. Conclusions

The acetate-CIMS provides a real-time measurement of gas-phase phenoxy herbicides with application to field studies. This case study demonstrates that both spray droplet volatilization and post-application direct volatilization are relevant mechanisms for phenoxy herbicides to enter the atmosphere, but that the relative importance of different atmospheric sources of herbicides likely depends on their chemical properties. Herbicides can enter the atmosphere through partitioning from spray droplets during the application process (spray drift volatilization), volatilization from the applied herbicide mixtures from soil and plant surfaces (short-term volatilization), and volatilization from the applied herbicides following ground-penetration (long-term volatilization). Both MCPA and 2,4-D can thus be easily and unintentionally transported to off-target areas via the atmosphere. 

The transport distance for atmospheric pesticides from application source to receptor site depends on wet and dry deposition rates, oxidation chemistry, and gas-particle partitioning. In the absence of precipitation events, a dry deposition velocity of 1.0 cm/s corresponds to a lifetime of ~29 h (assuming a typical boundary layer height of 1000 m), while using k_OH_ estimates for the two compounds corresponds to a lifetime of 16 h for MCPA and 28 h for 2,4-D, assuming average OH of 1.5 × 10^6^ molecules cm^−3^. While oxidation products of these compounds were not observed in the atmosphere during the short (<4 h) timescales of this experiment, longer post-application observation times would provide insight into the role of atmospheric oxidation chemistry in influencing the fate of applied herbicides, as well as the role of soil–air interactions [56] in controlling the atmospheric source of herbicides. This work focused on gas-phase herbicide concentrations and did not investigate partitioning to the aerosol phase, a process that can influence the atmospheric lifetime of pesticides [57]. Thus, while the gas-phase herbicide concentrations observed are not acutely toxic, volatilization sources warrant further consideration in both field measurement and modeling studies.

## Figures and Tables

**Table 1:** Chemical properties of observed herbicides. The K_OC_ is the soil adsorption coefficient and is soil dependent.

Compound	Chemical Formula	Chemical Form	Vapor Pressure (mmHg, 25 °C)	Henry’s Law Constant (atm m^3^/mole)	Water Solubility (mg/L)	K_OC_ (ml/g)
MCPA	C_9_H_9_ClO_3_	Dimethyl amine salt of 2-methyl-4-chlorophenoxyacetic acid	5.9 × 10^−6^ [44]	4.8 × 10^−10^ [45]	630 (25 °C) [46]	52–60 [47]
2,4-D	C_8_H_6_Cl_2_O_3_	2,4-dichlorophenoxyacetic acid	1.4 × 10^−7^ [48]	9.8 ×10^−8^ [48]	851 (25 °C) [45]	72–135 [49]
